# Evaluation of pathological complete response rates in breast cancer patients undergoing neoadjuvant therapy

**DOI:** 10.61622/rbgo/2025rbgo13

**Published:** 2025-04-30

**Authors:** Gabriella Ferezini Oliveira de Sá, Pedro Vilar de Oliveira Villarim, Pedro Hortêncio Saboia da Escossia Melo, Ayane Cristine Alves Sarmento, Ana Katherine Gonçalves, Kleyton Santos de Medeiros, Cristina Rocha de Medeiros Miranda

**Affiliations:** 1 Universidade Federal do Rio Grande do Norte Natal RN Brazil Universidade Federal do Rio Grande do Norte, Natal, RN, Brazil.; 2 Liga Contra o Câncer Nata RN Brazil Liga Contra o Câncer, Nata, RN, Brazil.

**Keywords:** Breast neoplasm, Neoadjuvant therapy, Surgical oncology

## Abstract

**Objective:**

This study aims to assess the rate of pathological complete response (pCR) in breast cancer patients undergoing neoadjuvant therapy and to explore its correlation with clinical, molecular, and prognostic factors.

**Methods:**

We conducted this retrospective observational study at Liga Contra o Câncer, a major public oncology reference center in Northeast Brazil. We included patients diagnosed with breast cancer who initiated neoadjuvant therapy between June 2018 and June 2019. Patients with a history of recurrent breast cancer or those who did not undergo surgery were excluded. The primary outcome was the pCR rate, with secondary outcomes including Overall Survival (OS), Disease-Free Survival (DFS), mortality, and disease recurrence. Follow-up extended until August 2022. We performed multivariate Cox regression analysis to correlate outcomes with predetermined variables.

**Results:**

Of the 292 included patients, 63 (21.6%) achieved pCR. The mean follow-up duration was 42.8 months. Multivariate logistic regression analysis revealed an association between pCR and the AC-TH regimen [OR = 2.4; 95%CI = 1.13 - 5.24; p=0.023], as well as between pCR and HER2-positive tumors [OR 2.49; 95% CI = 1.14 - 5.86; p=0.028]. Complete pathological response was associated with higher DFS [HR 0.33; 95%CI 0.13-0.86; p=0.024].

**Conclusion:**

Neoadjuvant therapy demonstrated significant efficacy in achieving pathological response in breast cancer patients. We observed a strong association between the AC-TH regimen, HER2-positive status, and pCR.

## Introduction

Breast cancer is the tumor with the most impact on morbidity and mortality in women worldwide. According to the World Health Organization (WHO), in 2020, there were an estimated 2.3 million new cases and 684,996 deaths, which represented 15.5% of cancer-related deaths in women.^(1)^ In this scenario, neoadjuvant therapy (NAT) reduces tumor volume and facilitates surgical resection. This approach aims to improve outcomes since it allows less extensive surgeries, fewer re-surgical excisions, and a reduction in positive margins during surgery.^(2,3)^

In addition, NAT can also provide information on the tumor's response to chemotherapy drugs, which contributes to more accurate therapeutic planning.^(4,5)^

One of the goals of NAT is to achieve a pathological complete response (pCR), defined as the absence of invasive carcinoma in the tissue removed during surgery.^(6)^ The pCR is a predictor of favorable long-term outcomes and is one of the parameters used by the Food and Drug Administration (FDA) in approval processes for new NAT regimens.^(7)^ However, literature data remain conflicting regarding the correlation between pCR and improved overall survival and disease-free survival outcomes, which reveals the importance of further studies.^(8)^

There is also no scientific consensus on the predictive factors most associated with pCR,^(9)^ which could help identify the populations that would benefit the most from neoadjuvant therapy.^(10)^

Brazil is a country marked by socioeconomic inequalities and a large part of the population depends on its public health system (Unified Health System—SUS)].^(11)^ Analyzing aspects of a treatment offered by this service becomes even more important, considering its inherent difficulties and limitations. Also, data on this type of treatment in Northeast Brazil is still scarce.

Thus, this study aims to evaluate the pathological complete response rate achieved by breast cancer patients undergoing NAT and to correlate this response with clinical, molecular, and prognostic factors.

## Methods

This retrospective observational study was conducted at Liga Contra o Câncer, a major public oncology reference center in Northeast Brazil, between June 2018 and June 2019, with follow-up through August 2022. The study was conducted in accordance with the Strengthening the Reporting of Observational Studies in Epidemiology (STROBE) statement.^(12)^

Patients eligible for inclusion were those over 18 years of age with invasive breast cancer confirmed by core biopsy who began neoadjuvant therapy between June 2018 and June 2019. Patients with a history of treatment for recurrent breast cancer, those with evidence of metastasis, or those with exclusively in situ tumors were excluded. Additionally, patients who did not undergo surgery and were considered not to have completed treatment were also excluded from the analysis.

Since this is a census study, wherein all medical records of patients meeting the inclusion criteria were analyzed and identified by a code indicating they had commenced neoadjuvant therapy, there was no need for a sample size calculation.

The neoadjuvant chemotherapy regimens used in this study included:

- Doxorubicin + Cyclophosphamide and Paclitaxel/Docetaxel (AC-T);
- Doxorubicin + Cyclophosphamide and Paclitaxel/Docetaxel + Trastuzumab (AC-TH);
- AC-T + Carboplatin;
- 5-fluorouracil + Doxorubicin + Cyclophosphamide (FAC);
- Other regimens selected by the attending physicians following the NCCN Clinical Practice Guidelines for breast cancer treatment (2016).^(13)^

The maximum treatment duration allowed by the public health system was six months. After completing neoadjuvant therapy, patients underwent surgery, with the specific procedure tailored to each individual case.

Data collected from the patient's electronic medical records included:

- Sociodemographic variables (age, sex, race, education);
- Anatomopathological characteristics (histological type and grade, nuclear grade, and lymph node involvement);
- Clinical characteristics (initial clinical staging, post-treatment pathological staging, pathological complete response (pCR), overall survival (OS), disease-free survival (DFS), disease progression during treatment, all-cause mortality, and treatment discontinuation);
- Neoadjuvant treatment data (surgical procedures, chemotherapy regimens, and drug adherence);
- Immunohistochemical characteristics of cell receptors: tumors with estrogen receptor (ER) positive and progesterone receptor (PR) positive were classified as luminal types A or B according to the Ki-67 proliferation index being < or ≥14. Human epidermal growth factor receptor 2 (HER2) expression was determined by immunohistochemistry or fluorescence in situ hybridization (FISH)/silver in situ hybridization (SISH) when necessary. If both hormone receptor and HER2 were positive, we classified the tumor as Luminal HER. Molecular subtypes were divided into five groups: luminal A, luminal B, luminal HER, HER2 overexpression, and triple-negative.

The tumor response to neoadjuvant therapy was assessed by comparing clinical staging data before treatment to pathological staging post-surgery. Pathological complete response was defined as the absence of residual invasive carcinoma in the breast and lymph nodes (ypT0N0 or ypTisN0). In the Residual Cancer Burden Index (RCB), they correspond to RCB 0.

All tumors that did not achieve pCR were classified as "not pCR", which, in the RBC index, corresponds to RBC I-III. Pathological partial response was defined as tumors that did not achieve pCR but regressed at least one stage in T and N or regressed in one classification while remaining stable in the other. Disease progression was characterized by an increase in T and/or N staging. Tumors with unchanged T and N staging were defined as stable diseases.

The primary outcome was achieving pCR at the pathologic staging after surgery, and the secondary outcomes were OS, DFS, mortality, and disease recurrence during the follow-up period. Data was analyzed using R 4.0.2 software (R Core Team). Categorical data were presented as frequency and percentage. The multivariate Cox regression model was used to calculate hazard ratios (HR) and their respective 95% confidence intervals for pCR, DFS, and OS. The alpha significance level was set at 5%. The Kaplan-Meier estimator was used to estimate recurrence-free and overall survival curves, with the log-rank test used to compare curves.

The multivariate analysis included variables such as pathological response, age at diagnosis, histological grade, clinical staging at diagnosis, clinical T and N staging at diagnosis, time between the last NAT cycle and surgery, presence of HER2 receptors, and drugs used during NAT.

The Liga Contra o Câncer ethics committee approved the study on July 13, 2022 (CAAE: 39393020.7.0000.5293). Data were collected from medical records; therefore, the ethics committee waived individual patient consent.

## Results

We reviewed 375 potentially eligible medical records. After individual evaluation, 19 records were excluded (5 were duplicates and 14 did not meet the inclusion criteria). We collected data from the remaining 356 records but excluded 64 because those patients did not undergo surgery. This left a total of 292 patients in the study. Table 1 shows the sociodemographic characteristics and treatment information for these patients.

**Table 1:** Sociodemographic characteristics, tumor, and treatment information

Variables		Sample size = 292 n(%)
Age, years, Median		52.5(23 - 87)
Race		
	Non caucasian	241(82.5)
	Caucasian	51(17.5)
Education		
	Elementary school	148(50.7)
	High School	89(30.5)
	University education	37(12.7)
	Without any	15(5.1)
	Not informed	3(1.0)
Clinical T staging (cT)		
	T1	4(1.4)
	T2	122(41.8)
	T3	114(39)
	T4	52(17.8)
Clinical N staging (cN)		
	N+	203(69.5)
	N0	89(30.5)
Clinical staging		
	I	2(0.7)
	II	139(47.6)
	III	151(51.7)
Histological grade		
	I	25(8.6)
	II	186(63.7)
	III	80(27.4)
	Not informed	1(0.3)
Molecular subtype		
	Luminal A	16(5.5)
	Luminal B	52(17.8)
	Luminal HER	123(42.1)
	HER2 overexpression	65(22.3)
	Triple negative	25(8.6)
	Not informed	11(3.8)
HER2		
	Positive	188(64.4)
	Negative	98(33.5)
	Not informed	6(2.1)
Drugs used		
	AC -T	231(79.1)
	ACT-H	50(17.1)
	FAC	3(1.0)
	AC-T + Carboplatin	2(0.7)
	Other	6(2.1)

The median was made with the age of breast cancer diagnosis; NA= Not applicable, NAT= neoadjuvant therapy, HER2= Human epidermal growth factor receptor 2, AC-T= Doxorubicin + Cyclophosphamide and Paclitaxel/Docetaxel, ACT-H= Doxorubicin + Cyclophosphamide and Paclitaxel/Docetaxel + Trastuzumab, FAC= 5-fluorouracil + Doxorubicin + Cyclophosphamide

Among the patients who received treatment, 63 (21.6%) achieved pCR. Of those who did not achieve pCR, 126 (43.2%) had a partial pathological response; 32 (11%) maintained stable disease; and 71 (24.2%) experienced disease progression. During follow-up, 63 patients (21.6%) had disease recurrence, while no recurrence was recorded for 229 (78.4%) up to the data collection cut-off. A total of 41 patients (14%) died during the follow-up period. The pCR rate varied according to the molecular subtype of the tumors analyzed. We found a pCR rate of 43.1% in HER2 overexpressed, 18.7% in Luminal HER, 0% in Luminal A, 1.9% in Luminal B, 32% in Triple Negative, and 2.7% in tumors with unreported molecular subtypes (Table 2).

**Table 2:** Assessment of pathological complete response by molecular subtypes

Molecular subtype	Total	pCR n(%)
HER2 Overexpression	65	28(43.1)
Luminal HER	123	23(18.7)
Luminal A	16	0(0.0)
Luminal B	52	1(1.9)
Triple negative	25	8(32.0)
Not informed	11	3(2.7)

pCR= Pathological Complete Response; HER2= Human epidermal growth factor receptor 2

Regarding the association between the analyzed variables with pCR, a multivariate logistic regression analysis showed that the use of the ACTH regimen was significantly associated with achieving pCR [OR = 2.43; 95% CI = 1.13 - 5.24; p = 0.023], as was the presence of HER2 receptor in tumor's immunohistochemistry [OR = 2.49; 95% CI = 1.14 - 5.86; p = 0.028] (Table 3).

**Table 3:** Multivariate analysis of clinicopathological factors associated with Pathologic Complete Response

Variables	n	OR	95% CI	p-value
Age at diagnosis				
< 50 years	23/105	—	—	
> 50 years	37/180	1.05	0.55, 2.04	0.9
Histological grade				
III	26/80	—	—	
I	0/24	0.00	0.00, 882690	> 0.9
II	34/181	0.41	0.21, 0.78	0.0007
Clinical Staging				
II	35/135	—	—	
I	1/2	12534859	0.00, NA	> 0.9
III	24/148	0.61	0.19, 1.92	0.4
Clinical T staging (cT)				
T4	8/49	—	—	
T1	1/3	0.00	NA, ∞	> 0.9
T2	31/120	1.36	0.37, 5.12	0.6
T3	20/113	1.03	0.38, 2.96	> 0.9
Clinical N staging (cN)				
N+	35/197	—	—	
N0	25/88	1.38	0.60, 3.16	0.4
Time between last NAT cycle and surgery				
More than 8 weeks	20/105	—	—	
Less than 8 weeks	40/180	0.82	0.60, 3.16	0.4
HER2				
Negative	10/98	—	—	
Positive	50/187	2.49	1.14, 5.86	0.028
Drugs used				
AC-T	36/227	—	—	
ACT-H	20/48	2.43	1.13, 5.24	0.023
Other	4/10	3.32	0.70, 15.0	0.12

OR* = Odds Ratio, CI** = Confidence Interval. NA= Not applicable, NAT= neoadjuvant therapy, HER2= Human epidermal growth factor receptor 2, AC-T= Doxorubicin + Cyclophosphamide and Paclitaxel/Docetaxel, ACT-H= Doxorubicin + Cyclophosphamide and Paclitaxel/Docetaxel + Trastuzumab

**Figure 1:**
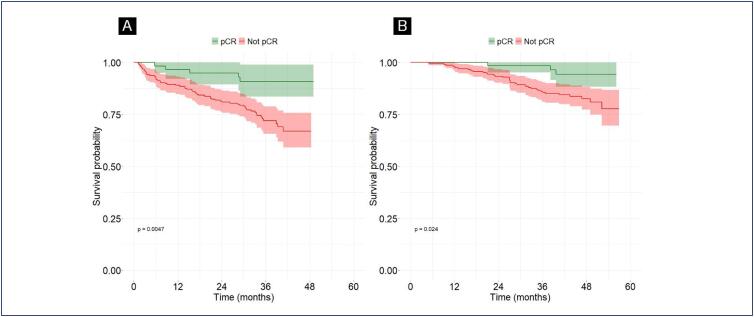
Kaplan-Meier survival curves obtained by the univariate analysis. A: Overall Survival in two subgroups defined by divergent tumor response to NAT: pCR / no pCR. B: Disease Free Survival in two subgroups defined by divergent tumor response to NAT: pCR / no pCR

The survival analysis was conducted with a mean follow-up time of 42.8 months. The 3-year OS rate was 89%, and the 3-year DFS rate was 76%. Regarding the Kaplan Meier Curve designed with the univariate analysis data, it was found a significant association between pCR and OS, and pCR and DFS (Figure 1).

However, the multivariate analysis suggests only a significant association between achieving pCR (RCB 0) and improved DFS compared to not achieving pCR (RCB I-III) (HR = 0.33; 95% CI = 0.13 - 0.86; p = 0.024). In the multivariate analysis, pCR was also associated with greater OS, but without statistical significance (HR = 0.34; 95% CI = 0.10 - 1.18; p = 0.091). Additionally, clinical stage III at diagnosis was associated with lower OS compared to stage II (HR = 4.95; 95% CI = 1.14 - 21.4; p = 0.033). A total of 69 patients did not complete the prescribed NAT before surgery. Of these, 25 could not finish treatment within the 6-month timeframe allowed by the Brazilian public health system; 20 discontinued NAT due to chemotherapy-related toxicity; 13 stopped because of disease progression; 3 abandoned treatment; and 8 did not complete NAT for other reasons.

## Discussion

The results obtained in this research showed that neoadjuvant treatment was highly effective in inducing a pathological response among breast cancer patients. Additionally, a strong correlation was observed between the AC-TH regimen, HER2-positive status, and achieving pCR.

The pCR rate observed was higher than in previous research, such as a study at another Brazilian medical center, that reported a pCR rate of 17.1%, and a systematic review by Mauri et al.^(14)^ that found pCR rates from 4% to 29%.^(14-16)^ This suggests that our treatment approach may be effective, despite the challenges of the Brazilian public health system, including financial constraints, social factors, and limited resources.

When comparing pCR rates across different molecular subtypes, it was observed that HER2-negative tumors represented a small proportion of tumors with pCR.^(17)^ This can be partially explained by the high prevalence of HER2-positive tumors in our sample (64.4%) compared to the expected distribution of molecular subtypes in breast cancer.^(18)^ Due to this selection bias, the assessment of the response stratified by the other molecular subtypes was limited.

Similar to previous studies, our results showed that HER2-positive patients have a greater sensitivity to NAT, with higher rates of pCR.^(6,19)^ The strong correlation between the use of Trastuzumab and increased pCR rates suggests that this drug should be considered more frequently for HER2-positive patients. Since HER2 positivity is often associated with more aggressive cancer and poorer prognosis, initiating targeted therapy during NAT, rather than waiting until adjuvant treatment, may lead to better outcomes.^(6)^

Despite these favorable results for anti-HER2 targeted therapy in our study, only 50 out of 188 presenting this immunohistochemical marker received the treatment. This data can be related to the Brazilian public health system bureaucracy to obtain Trastuzumab, which complicates access to a more effective treatment. Due to the system's budgetary limits, we notice a delay in the inclusion of effective, but costly, medications in the therapeutic arsenal. It is also noticed a delay in dispensing targeted therapies, which could take weeks to months and harm the results of the proposed treatment strategy. This means that a significant number of patients who could benefit from treatment are not covered and may be associated with a worse prognosis in the disease progression.

Regarding clinical outcomes, our results show that pCR was significantly associated with improved DFS, which is consistent with previous findings.^(20-23)^ This further emphasizes the value of NAT in determining the tumor's response to specific therapeutic strategies, allowing for more informed decisions on adjuvant treatments.^(4,5)^ However, the association between pCR and OS was not significant, contrary to other studies, as reported by Mackelenbergh MT et al. and Huang et al., that found an association between long-term survival and pCR achieved in NAT.^(20,21)^ Possibly, these findings weren't observed in our study, due to the limited sample size, short follow-up time, and methodological limitations.

Additionally, our results suggest that patients diagnosed at clinical stage II had better OS than those at stage III, indicating that early diagnosis could lead to improved outcomes.^(24)^ Our sample included a significant proportion of patients under 50 years with locally advanced disease at diagnosis, suggesting a trend of late diagnosis in Brazil, possibly due to the screening cut-off age of 50.^(25)^ This raises the question of whether earlier screening could lead to better outcomes.^(26)^

Our study presents some limitations due to its observational and retrospective nature. It was not possible to control and standardize the indications for NAT in the studied population and the treatment regimen used, and indications for neoadjuvant therapy could have been influenced by different attending physicians. Inconsistencies of this nature may impact analyses concerning the association between pCR and different treatment modalities.

Also, the study is subject to the loss of potentially relevant data. There was a lack of information in the medical records of some patients regarding their health status at the time the data was collected, either due to abandonment of the service or due to unreported mortality, which was probably aggravated by the Coronavirus Disease 2019 (COVID-19) pandemic. This contributed to the fact that follow-up time was different among all patients, which may have influenced the OS results leading to the absence of statistical significance in multivariate analysis.

To minimize the biases of an observational and retrospective study, multivariate analysis was performed to acquire independent associations between studied variables. To mitigate the recall bias associated with the retrospective study with information obtained from medical records, the data collection carried out by the researchers used objective parameters that did not depend on the subjectivity of the attending physician, as well as hard outcomes to evaluate the response to treatment.

Clinical trials involving a larger participant pool and longer follow-up time are necessary to yield more robust and reliable results. However, this study contributes to new knowledge to the scientific community by providing relevant data on a type of treatment that is highly dependent on the public health system in Brazil. It indicates that neoadjuvant treatment seems to improve breast cancer patient prognosis, despite the limitations imposed by a system that hinders its full implementation.

## Conclusion

NAT demonstrated significant results in terms of pathological response. Additionally, we observed a strong correlation between the AC-TH regimen in neoadjuvant therapy, HER2-positive patients, and complete pathological response.
